# Measuring Parental School Involvement: A Systematic Review

**DOI:** 10.3390/ejihpe15060096

**Published:** 2025-05-31

**Authors:** Helena Mocho, Cátia Martins, Rita dos Santos, Elias Ratinho, Cristina Nunes

**Affiliations:** University Research Center in Psychology (CUIP), University of Algarve, 8005-139 Faro, Portugal; hsalcaparra@ualg.pt (H.M.); rasantos@ualg.pt (R.d.S.); jeratinho@ualg.pt (E.R.); csnunes@ualg.pt (C.N.)

**Keywords:** children, measures, parental school involvement, parents, psychometric, systematic review

## Abstract

Parental school involvement (PSI) is a multidimensional construct that significantly influences children’s academic adjustment and overall development. However, inconsistencies persist in its definition and measurement, with researchers operationalizing PSI through a varied of parental activities. This study aimed to (1) identify PSI instruments and their theoretical models and (2) evaluate their psychometric properties. Following PRISMA guidelines (PROSPERO ID CRD42023451091, registered August 2023), this systematic review examined six databases (Web of Science, ERIC, MEDLINE, Psychology and Behavioral Sciences Collection, PsycArticles, and PsycInfo), analyzing quantitative studies from 2000 to 2024. Inclusion criteria encompassed instruments designed for parents, teachers, or children aged 6–15 and published in peer-reviewed journals in English, Portuguese, Spanish, or French. From an initial pool of 490 records, 38 studies were included, yielding 43 instruments: 23 for parents, 15 for children, and 5 for teachers. Seven instruments followed Epstein’s model, while nine followed the Hoover-Dempsey and Sandler framework, underscoring the prominence of these theoretical approaches. The study quality, assessed with MMAT, was generally moderate to high. Despite an exhaustive search effort, it is possible that some relevant instruments were not identified. Nevertheless, this review advances the understanding of PSI operationalization, promotes more consistent and replicable assessments, enhances alignment between instruments and study objectives, and strengths the validity of findings derived from these tools.

## 1. Introduction

Parental school involvement (PSI) refers to parents’ participation in their children’s educational processes and experiences (Jeynes, 2005; LaRocque et al., 2011). It comprises a range of activities within both family and school contexts, typically categorized into three domains: (1) home-based involvement, (2) school-based involvement, and (3) home–school communication (Ma et al., 2022). PSI serves as both a determinant of learning and a protective factor against academic and social challenges (Boonk et al., 2018; Castro et al., 2015; Hornby & Blackwell, 2018; Lerner et al., 2022; Oswald et al., 2018). As such, it is a multidimensional construct that plays an important role in shaping children’s academic adjustment and broader developmental outcomes (Barger et al., 2019; Boonk et al., 2018; Castro et al., 2015; Epstein & Sanders, 2002; Mocho et al., 2024).

PSI has been conceptualized in diverse ways (Chen & Zhu, 2017). Some scholars adopt a broader perspective that includes parental investment across social, emotional, and behavioral domains (Castro et al., 2015), while others focus on specific involvement strategies (e.g., home-based or school-based activities) (Boonk et al., 2018; Perrigo et al., 2023). One of the most influential frameworks is Epstein’s (2010), which outlines six key types of involvement: (1) parenting (supporting a home environment conducive to learning), (2) communicating (maintaining school–family dialogue), (3) volunteering (participation in school activities), (4) learning at home (helping children with homework and academic tasks), (5) decision making (engaging in school governance), and (6) collaborating with the community (connecting external resources to school). Fisher (2011) further expanded this by identifying 44 PSI-related activities classified by location (inside/outside school), organizational level (individual/institutional), and focus (resources, control, pedagogy, and well-being).

A meta-analysis identified several psychological and behavioral predictors of PSI, such as parental self-efficacy and perceived school invitations, which influence parental willingness to engage and are linked to children’s academic and psychological development (Barger et al., 2019). Considerable attention has been given to understanding the motivational factors that underpin PSI (Jay et al., 2017). Of particular interest is autonomy-supportive involvement, which has been associated with enhanced academic outcomes and greater student motivation (Lerner et al., 2022).

PSI positively impacts both families and children. For parents, it strengthens relationships with teachers and enhances confidence, satisfaction, and interest in their own educational roles (e.g., Hornby & Blackwell, 2018). For children, it contributes to a more positive school climate, improved attendance, better attitudes toward school, and positive behavioral and mental health (e.g., Antipkina & Ludlow, 2020; Hornby & Blackwell, 2018).

PSI is underpinned by both psychological and behavioral components that promote children’s academic and socioemotional well-being (Cheung & Pomerantz, 2011; Pomerantz & Monti, 2015). Alongside PSI, other school climate variables—such as supportive teacher–student relationships, extracurricular opportunities, and peer interactions—foster students’ sense of belonging (El Zaatari & Maalouf, 2022). This sense of belonging significantly influences academic, behavioral, and psychological functioning (Štremfel et al., 2024). However, dysfunctional forms of PSI may predict academic and emotional problems (externalizing and internalizing), including poor performance, increased delinquency or cyberbullying, and elevated depression and anxiety (e.g., Barger et al., 2019; Boonk et al., 2018; Guo et al., 2022).

Parents’ self-motivation is a key factor in their engagement. Self-Determination Theory (Ryan & Deci, 2017) posits that parental involvement may stem from various motivations, ranging from external pressures (e.g., school demands) to introjected motivates (e.g., fear of being perceived as a “bad parent”), identified motivation (valuing the behavior), or intrinsic interest (enjoyment) (Grolnick, 2015; Lerner et al., 2022).

PSI evolves as children grow (El Nokali et al., 2010; Wang & Sheikh-Khalil, 2014). In early childhood, teachers often play a central role in supporting emotional development, and collaboration with parents is vital (Lui et al., 2020; Kartel et al., 2022; Sanz-Ponce et al., 2023). As children mature, PSI tends to shift from direct involvement to fostering motivation and setting academic expectations (Boonk et al., 2018; Choi et al., 2015). Differences in how parents and teachers perceive their roles can cause misunderstandings that affect children’s academic progress (Howard et al., 2023), highlighting the importance of coordinated support within the educational triad—student, parents, and teacher (Martín Retuerto et al., 2020). Despite these challenges, parental support remains crucial, underscoring the need for valid instruments to assess PSI in its various forms (Fan & Chen, 2001; Hill & Tyson, 2009; Ma et al., 2022).

A major challenge in PSI research is the lack of consistent definitions and measures. Because PSI is conceptualized differently across studies, researchers have operationalized it using different sets of activities and components from the broad set of aspects that describe this ill-defined construct (Antipkina & Ludlow, 2020; Wilder, 2014). Consequently, although measuring PSI is essential, it remains a complex task.

Another gap in the literature is the limited focus on PSI during elementary school. Most research has concentrated on adolescents, despite the likelihood that family dynamics and involvement patterns differ significantly during earlier developmental stages (Eccles, 2007; Guo et al., 2022). Moreover, few PSI measures targeting primary education populations have been published (Antipkina & Ludlow, 2020).

To advance PSI assessment, it is crucial to (1) ensure convergent and discriminant validity of efficient retrospective instruments involving parents, teachers, or children; (2) explore methods; and (3) evaluate PSI in the context of school initiatives such as parent–teacher meetings (Pomerantz & Monti, 2015). Additionally, tools that differentiate types of involvement and measure related engagement constructs are needed. Assessing both engagement behaviors and participation barriers is key to a comprehensive understanding of PSI.

Accurate measurement of PSI is essential for understanding its relationship with students and informing evidence-based strategies (Schueler et al., 2017). Although previous systematic reviews have addressed PSI in specific contexts (e.g., Acar et al., 2021; Avvisati et al., 2010; Boonk et al., 2018; Gülhan, 2023; Hingle et al., 2010; Yang et al., 2023), none have focused specifically on the psychometric qualities of PSI measures. By identifying instruments and their theoretical models (Mettert et al., 2020), this systematic review (SR) aims to reduce measurement heterogeneity and improve the operationalization of PSI.

Thus, the objectives of this study are to (1) identify PSI instruments and their theoretical models and (2) examine their psychometric properties.

## 2. Method

### 2.1. Study Type and Research Objectives

This study is an SR that is defined by its methodical, comprehensive, transparent, and replicable methodology and presentation. SRs involve an exhaustive search for relevant research on a given subject, a systematic integration, and a critical appraisal of the extent, nature, and quality of the available evidence addressing the defined research question (Siddaway et al., 2019).

This SR followed a framework-based review approach. When grounded in established models, reviews tend to be more impactful, robust, and useful, as they allow for the extraction of key insights, identification of research gaps, guidance for future research, and integration of diverse literature streams (Paul et al., 2024). Overall, the objectives of this SR were to (a) provide an overview of studies assessing PSI, (b) report the psychometric properties of the instruments used, (c) analyze the theoretical models underlying these instruments, and (d) assess the methodological quality of the included studies.

### 2.2. Procedures

This SR was conducted in accordance with the PRISMA 2020 guidelines (Preferred Reporting Items for Systematic Reviews and Meta-Analyses; Page et al., 2021) to ensure a rigorous and transparent process.

#### 2.2.1. Research Questions

This SR aimed to answer the following research questions:

1. What instruments have been used to assess PSI with children aged between 6 and 15 years old?
2. What theoretical models underlie these PSI assessment instruments?

#### 2.2.2. Research Protocol and Registration

A detailed research protocol was developed prior to the review to reduce potential bias and ensure objectivity. The protocol was registered in PROSPERO, an international database of systematic reviews, on 14 August 2023 (ID no. CRD42023451091). Registering the protocol enhances the transparency, visibility, and replicability of the review, while also helping to prevent duplication of effort (Vilelas, 2022).

#### 2.2.3. Data Collecting

The literature search was conducted on 9 January 2025 in the following databases: Web of Science, ERIC, MEDLINE, Psychology and Behavioral Sciences Collection, PsycArticles, and PsycInfo. Studies between 1 January 2000 and 31 December 2024 were considered. The start date was selected due to the increased global focus on children’s academic success since 2000 following the publication of international assessment results such as the Trends in International Mathematics and Science Study and the OECD’s Programme for International Student Assessment, which have highlighted disparities and quality issues in education across OECD countries (CNE, 2017).

#### 2.2.4. Eligibility Criteria

The eligibility criteria were defined on the PICOST framework as presented in Table 1 (Population, Interventions, Comparators, Outcomes, Study design, and Time): (P) studies involving PSI in children aged between 6 and 15 years (grades 1–9) as well as parents and/or teachers; (I) quantitative studies; (S) sources and languages included peer-reviewed journal articles published in English, Portuguese, Spanish, and French; and (T) studies published between January 2000 and December 2024.

The decision to include only peer-reviewed journal articles was based on the expectation of greater methodological rigor and result reliability, reduced publication bias, and adherence to established scientific standards. Gray literature and conference abstracts were excluded due to their typically limited peer review and potential to present incomplete or preliminary findings, which may compromise replicability and transparency.

Additionally, studies that lacked psychometric validation were excluded due to concerns regarding measurement reliability and the integrity of synthetized evidence. This exclusion aligns with best practices in SRs, ensuring a high-quality, evidence-based synthesis.

The methodological quality of the included studies was assessed with the Mixed Methods Appraisal Tool (MMAT; Version 2018). The MMAT is designed for appraising the quality of mixed-studies reviews and includes a concise set of key criteria, allowing for efficient and reliable quality evaluation (Hong et al., 2018). The following types of studies were excluded: (a) books, literature reviews, or academic works; (b) studies focusing exclusively on specific characteristics or contexts of PSI (e.g., PSI in digital learning environments); (c) qualitative studies that were not eligible for MMAT assessment (Version 2018) (Hong et al., 2018); and (d) studies deemed low quality, with an MMAT final score below 50%).

#### 2.2.5. Information Sources and Search Strategy

The literature search was conducted in January 2025 using the following international scientific databases: Web of Science, ERIC, MEDLINE, Psychology and Behavioral Sciences Collection, PsycArticles, and PsycInfo. The following search strategy was employed: Parental involvement OR Parental engagement (Title) AND Measure OR Instruments OR Psychometric OR Assessment AND School AND children OR child OR teenagers OR adolescents OR parents OR mothers OR fathers OR teachers (Abstract). Also searched: Parent* involvement OR Parent* engagement (Title) AND Parent* involvement OR Parent* engagement (Abstract) OR Measure OR Instrument* OR Psychometric OR Assessment AND School AND child* OR teenag* OR adolesc* OR parent* OR mother* OR father*. The strategy was tailored to each database when necessary (Table A1; Appendix A).

#### 2.2.6. Study Selection

All search results were exported to an Excel spreadsheet, and duplicates were removed. Relevance was initially assessed by screening the titles and abstracts of the retrieved records. Potentially eligible studies were subjected to full text review to confirm whether they met the inclusion criteria. Studies that did not meet the criteria were excluded.

Study organization and management were supported by Rayyan Intelligent Systematic Review, an online tool designed to streamline SR and meta-analysis procedures (Johnson & Phillips, 2018). Rayyan is noted for its ease of use and its ability to reduce screening time and workload (F. Yu et al., 2022).

All data extraction and study screening were conducted independently and blindly by two researchers. After reaching full agreement on inclusion or exclusion decisions, the screening process continued with a single researcher, who was responsible for flagging any uncertainties. Final decisions on ambiguous cases were made by consensus among three researchers.

#### 2.2.7. Qualitative Assessments of the Studies

To assess the methodological quality of the included studies, we used the MMAT (Hong et al., 2018). This tool covers five categories: qualitative research, randomized controlled trials, non-randomized studies, quantitative descriptive studies, and mixed-method studies. Each study is evaluated against relevant criteria and scored as “Yes” (1 point) or “No” or “Can’t say” (0 points), resulting in a total score ranging from 0 to 5 points or 0 to 100%, which was the selected form of presentation for this SR.

Since the MMAT does not define cut-off values, the following classification system was adopted: (1) low-quality studies, for scores less than 50%; (2) medium-quality studies, for scores between 50% and 80%; and (3) high-quality studies, with scores above 80%. The lowest-quality studies were excluded from the final sample.

The quality assessment followed a similar procedure to the data-screening process. The first ten studies were independently assessed by two researchers. As their evaluations were in full agreement, the remaining studies were assessed by a single researcher. At the end of the assessment, three researchers convened to review studies with potentially concerning quality and reach a consensus. Table A2 (Appendix B) presents a summary of each included study, including its quality score, objective, main findings, and limitations/strengths.

#### 2.2.8. Data Extraction

Data extraction was conducted systematically for each study. The following information was collected: database, author(s), article title, abstract, publication date, language, country, type of study, aim(s), sample, instrument name, instrument type, original authors, instrument date, subscales, number of items, number of response options, reading level required, administrations length, Cronbach’s alpha, instrument notes, theoretical model, validity, results, limitations, suggestions, conclusions, construct definition included, content validity analysis, statistical analysis of items, external validity analysis (criterion, convergent, discriminant), exclusion of SR (yes/no), reason for exclusion, and observations/notes.

## 3. Results

### 3.1. Descriptive Characteristics

The initial database search yielded 490 peer-reviewed articles. After identifying and removing duplicates (*n* = 225 studies), 154 records were excluded based on their title and summary. The remaining 111 articles were reviewed in full, and an additional 8 studies were identified through snowballing techniques.

Several studies were excluded for methodological reasons to maintain scientific rigor. For example, Guo et al. (2022) was excluded because it measured different PSI characteristics using subscales from multiple instruments originally developed by Walker et al. (2005), which are already in this SR. Jhang’s (2019) study was excluded as it employed the Pingtung Education Longitudinal Survey, a tool that, despite covering many dimensions of PSI, is not exclusively focused on the PSI construct. Similarly, the Green et al. (2007) study was not included because it addressed only the first level of Hoover-Dempsey and Sandler’s (2005) revised theoretical model (Walker et al., 2005).

Regarding the qualitative assessment of the final 38 studies included in this SR, the MMAT scores were as follows: 22 studies scored 100% [2–7, 11–15, 17, 19, 21–23, 25, 26, 29, 34, 36–41, 43], 14 studies scored 80% [1, 8–10, 16, 18, 24, 27, 28, 30–32, 35, 42], and 2 scored 60% [20, 33]. These scores range from moderate to excellent, and no studies were excluded based on the MMAT criteria (Appendix B; Table A2).

**Figure 1:**
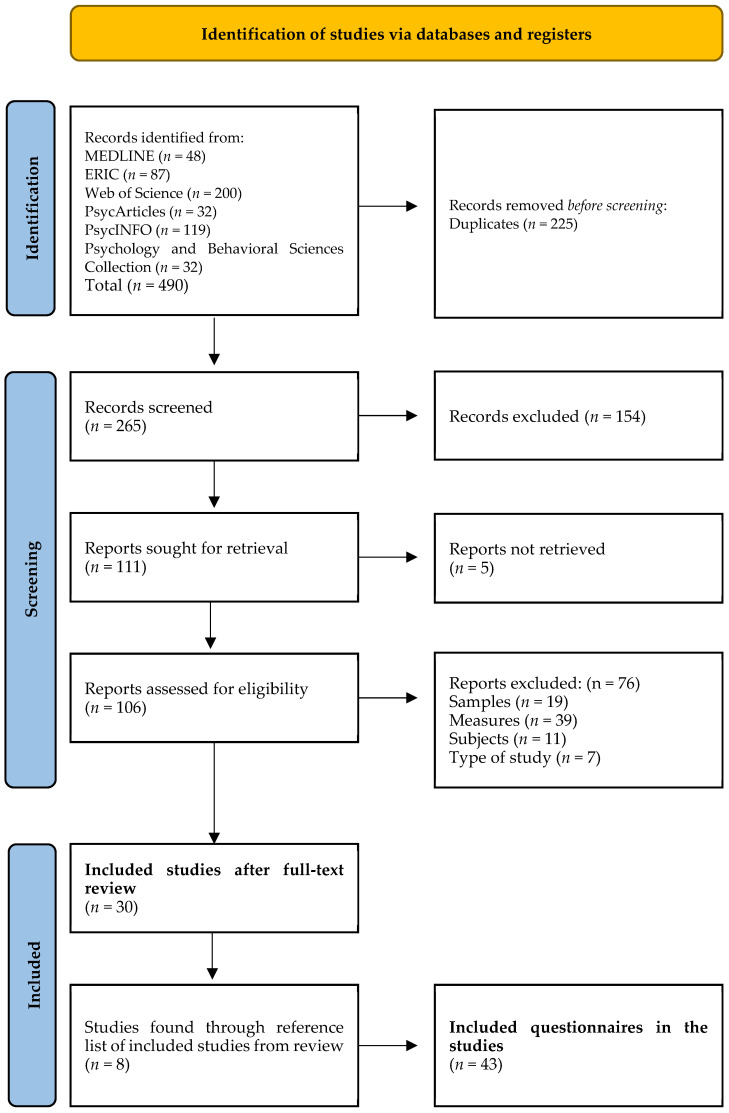
Systematic review flow diagram.

In total, 38 articles and 43 quantitative instruments were included in this SR (Figure 1). Descriptive details of the studies and instruments are presented in Table 2, while their theoretical models and psychometric properties are listed in Table 3.

**Figure 2:**
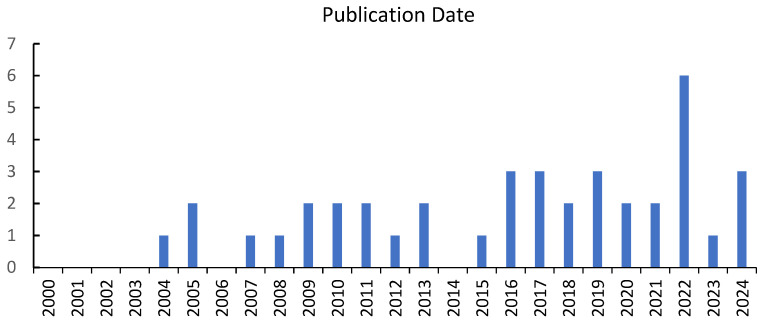
Studies’ year of publication.

Regarding publication years, three notable gaps were identified: 2000–2003, 2006, and 2014 (Figure 2), during which only one to three studies were published. A slight increase was observed in 2022, with six articles published (*f* = 6). Analysis by country (*f* = 20) revealed that the United States led with the highest number of studies (*f* = 8), followed by China, Nigeria, and Spain (*f* = 3 each).

Overall, 43 instruments were identified in the included studies, of which 23 were completed by parents [2, 4–8, 11, 12, 15, 16, 18, 23, 25, 27–29, 31, 32, 34–37, 40], 15 by children [3, 9, 10, 14, 17, 19, 20–22, 24, 26, 30, 33, 39, 42], and 5 by teachers [1, 13, 38, 41, 43]. In terms of instrument subscales, the number ranged from 1 to 8 (*M* = 3.74; *SD* = 1.64); however, five instruments did not report subscale information.

The number of items ranged from 4 to 72 (*M* = 24.88; *SD* = 15.26; *Mo* = 10.00; *Md* = 22.00), with two studies not reporting this information [18, 28]. Most instruments used a Likert-type scale ranging from 1 (e.g., never, strongly disagree, not true) to 6 (e.g., always, strongly agree, very true). Only one study failed to report the range of response options. None of the studies identified the administration time or the required reading level for completing the instrument.

Sample sizes across the selected studies ranged from 24 to 17,563 individuals (*M* = 1481.94; *SD* = 3351.83; *Mo* = 155.00; *Md* = 496.00). More specifically, for studies involving parents, sample sizes ranged from 155 to 17,563 individuals (*M* = 2176.00; *SD* = 4426.88); for children, sample sizes ranged from 120 to 1895 (*M* = 750.06; *SD* = 681.41); and for teachers, sample sizes ranged from 24 to 1364 (*M* = 353.60; *SD* = 567.28).

With respect to the language of the instruments (Table 2), the majority were available in English [e.g., 4–6, 12–14, 23, 25, 27, 32], followed by Chinese [14, 17, 21, 35, 36, 42]. Additionally, four instruments were in Spanish [7, 22, 26, 30], two in Turkish [11, 15], and two were in Portuguese [1, 8].

### 3.2. Theoretical and Psychometric Characteristics

The underlying theoretical models (Table 3) varied across the instruments. Specifically, 7 instruments were based on Epstein’s (2010) [1, 2, 7, 8, 32, 37, 38]; 9 were based on Hoover-Dempsey and Sandler’s (1995) model [4–6, 15, 19, 25, 40, 41, 43]; and the remaining 27 instruments did not explicitly reference a theoretical model in their development or validation processes.

Among the instruments based on Epstein’s model, three were designed for teachers [1, 38, 41] and three shared the same subscale designations [32, 37, 38]. Regarding those aligned with the Hoover-Dempsey and Sandler model, three were used closely in the same study [4, 5, 6].

For instruments that did not identify a theoretical model, some inferences could be made. For instance, the instrument developed by Antipkina and Ludlow (2020) [31] utilized a Rasch/Guttman scenario-based scale, reflecting a holistic approach measuring PSI. Other instruments, although not explicitly referencing a model, used dimensions that suggest the theoretical underpinnings. For example, the Parental Involvement at School scale [28] is derived from the Parent Survey of Family and Community Involvement in the Elementary and Middle Grades (Sheldon & Epstein, 2007), which is rooted in Epstein’s model. Similarly, the Turkish Parental Involvement Scale [11] appears to be grounded in Epstein’s dimensions.

In terms of theoretical model distribution by respondent group, the results showed the following: 11 instruments for parents (25.6%), 4 instruments for teachers (9.3%), and only 1 (2.3%) used with children had a clearly identified theoretical framework.

As expected, the subscales of the instruments reflected the theoretical model upon which they were based. Instruments linked to Epstein’s model typically included dimensions such as parenting, communicating, volunteering, learning at home, decision making, and collaborating. Regarding the instruments associated with Hoover-Dempsey and Sandler’s model, the dimensions focused on home-based involvement, school-based involvement, or parents’ reports of their involvement and parents’ reports on invitations from teachers to be involved. For instruments without a theoretical model, recurring dimensions included cognitive involvement, behavioral involvement, personal involvement or parental pressure, parental psychological support, parental monitoring, resources for intellectual development, parental help, and parental participation in school.

With respect to psychometric properties (Table 3), 11 instruments reported content validity analysis [1, 2, 8, 11, 22, 23, 25, 29, 31, 34, 39], and 14 presented a clear construct definition [1, 2, 4–6, 8, 11, 18, 23, 25, 30, 31, 34, 39]. Twelve of the instruments reported item-level statistical analysis [1, 2, 8, 11, 14, 16, 22, 23, 25, 31, 34, 39].

Regarding dimensionality analysis, 1 instrument applied the Rasch rating scale model (performed with a sample of parents) [31], 4 instruments used Exploratory Factor Analysis (EFA) [1, 8, 28, 39], 10 conducted a Confirmatory Factor Analysis (CFA) [14, 16–17, 22, 25–26, 29, 34, 36, 42], 4 instruments used both EFA and CFA [2, 11, 21, 23], and 24 did not report any dimensionality analysis [3, 4–7, 9, 10, 12, 13, 15, 18–20, 24, 27, 30, 32, 33, 35, 37, 38, 40, 41, 43].

In terms of reliability estimation, most of the studies reported Cronbach’s alpha. Among instruments for parents, the alpha coefficients ranged from 0.53 to 0.97 [1, 4–8, 11, 12, 15, 16, 18, 23, 25,28, 29, 31, 32, 34–37, 40]; one of the instruments did not report reliability [27], and one instrument presented its reliability estimation with the McDonald’s omega coefficient (ranging from 0.74 to 0.82, and the total score was 0.92) [36]. In the children’s instruments, the alpha ranged from 0.65 to 0.96 [3, 9, 10, 14, 17, 19, 20–22, 24, 26, 30, 33, 39, 42]. Finally, teachers’ reliability ranged between 0.63 and 0.98 [2, 13, 38, 41, 43].

Despite of this, 35 studies (out of 38) did not provide any evidence of validity beyond reliability. Among the seven studies that did, the types of validity were discriminant (*f* = 5), convergent (*f* = 4), or criterion (*f* = 5).

For a more comprehensive understanding of the instruments, the objectives, strengths, and limitations of the studies included in this SR were also examined (see Appendix B). Most studies aimed to develop PSI instruments across different cultural and educational contexts, often analyzing psychological factors, socioeconomic background, school environment, and their impacts on children’s academic and emotional adjustment. The most frequently reported limitations involved the sample characteristics and the resulting limited generalizability of the results.

### 3.3. Best-Rated Instruments According to the Identification of the Theoretical Model and Principal Psychometric Characteristics

Following the analysis of the theoretical models and psychometric properties of the instruments included in this study, we identified a subset of instruments that demonstrated the most robust characteristics. This selection was based on three criteria: identification of the theoretical model, dimensionality analysis, and reliability estimation (Table 4). To be considered among the best-rated instruments (Table 4), an instrument had to meet at least two of the three defined criteria. Additionally, to support researchers and practitioners in choosing the most appropriate tool for their specific research objective, we included relevant descriptive information for each selected instrument, such as the number of items, language, and target population.

The prioritization of these three variables is grounded in their importance for ensuring both conceptual coherence and feasibility in psychometric analysis, particularly when compared with other, less consistently reported properties (e.g., validity evidence). This approach enhances the comparability, replicability, and interpretability of findings derived from different instruments.

Moreover, the restriction to widely reported and standardized characteristics ensures a more representative dataset and enables a more equitable comparison between instruments. By reducing the risk of exclusion bias related to incomplete reporting, this procedure contributes to the selection of methodologically sound and contextually appropriate instruments for assessing parental school involvement. The prioritization of these variables is based on the need to ensure consistency in identification and feasibility in data analysis compared with other psychometric characteristics. This approach allows for more robust and reliable results. The restriction to the selection of these three criteria, which are widely reported in the description of the instruments, ensures a more representative dataset. Furthermore, the standardization of the instrument selection criteria facilitates more equitable comparisons and minimizes potential exclusion biases.

## 4. Discussion

In this SR, our aim was to examine the available measures of parental school involvement (PSI), identify the theoretical models that undergird each measure, and review the psychometric properties of these instruments. Psychometric tools are expected to validly, reliably, and fairly represent the underlying construct they measure to be considered unbiased and accurate (Howard et al., 2023; Kristjansson et al., 2003).

Regarding our first and second aims, a total of thirty-eight articles were selected after analyzing the identified literature. This selection contributes to a comprehensive understanding of PSI and to the development of effective strategies for fostering involvement and evaluating the perspective of parents, children, and teachers regarding their participation as well as the perceived barriers to increased engagement or collaboration. Measuring such concepts remains challenging, particularly due to the uncertainty of whether a set of barrier items should function as a traditional scale—an issue compounded by parental time constraints (Schueler et al., 2017).

The results revealed an increase in publications in 2022. This could be partially explained by the scope of our SR, which excluded PSI measures that focused on (a) specific types of involvement (e.g., home-based activities only), (b) specific academic subjects (e.g., reading, math, or sports), or (c) particular child characteristics (e.g., attention-deficit/hyperactivity disorder or autism spectrum disorder). A broader review of the literature indicates that PSI remains a consistently relevant topic, driven by both interest in its potential benefits and the need to develop diverse tools to analyze this collaborative dynamic (e.g., Alieva, 2021; Eden et al., 2024). Additionally, interest may have intensified due to the shifts in PSI during and after the COVID-19 pandemic—a period that placed unprecedented demands on the home–school relationship, making home-based involvement critical, as parents assumed a more central role in the learning process (Raguindin et al., 2021).

A disparity in sample sizes among parents, children, and teachers was observed across the selected studies, raising questions about the generalizability of results. These discrepancies likely stem from the varied objectives and the instruments used across the studies, which in turn may have influenced their outcomes. Inadequate sample selection can lead to low precision and accuracy, compromising the quality of results derived from participants’ responses (El-Den et al., 2020).

Concerning the instruments reviewed, most lacked strong supporting evidence, particularly in terms of validity and dimensional analyses. For instance, even when replication studies adhered to rigorous statistical standards, their findings remained limited in value if no evidence of construct validity was presented (Flake et al., 2022). These results are in line with Wittkowski et al. (2020), who highlighted the insufficient evidence supporting the reliability of self-report instruments assessing parent–child relationships. As a result, we recommend cautious use of these tools. The meta-scientific literature advises against employing scales with little or no reported validity evidence (Flake et al., 2022; Slaney, 2017).

The findings of this SR support the broader critique regarding the challenges of operationalizing PSI and highlight the study’s relevance. Based on these results, researchers may evaluate the utility of existing instruments and conduct further research into their internal structures to identify and develop new dimensions or features. Future work might also involve updating these tools to reflect pedagogical innovations, including the integration of Information and Communication Technologies (ICTs) in schools and the evolving forms of teacher–parent communication. However, given the growing international and diffuse nature of the PSI literature, it is vital to possess both theoretical and analytical expertise to meaningfully synthesize and contextualize the available evidence (Goldstone et al., 2023).

Although the theoretical models of the reviewed instruments were not always explicitly detailed, their subscales and dimensions often reflect the underlying theoretical foundations. Still, the heterogeneity in how PSI is operationalized is compounded by inconsistent theoretical conceptualizations. PSI’s multidimensionality has led to definitional disagreements and measurement inconsistencies, making comparisons across studies difficult. While prior research has largely focused on how various PSI types relate to school outcomes, these forms of involvement often interact with one another (Hill & Tyson, 2009). The disconnect between theoretical definitions and empirical measurement ignores the interdependent nature of PSI constructs (Walker et al., 2005). As Walker et al. (2005, p. 100) noted: “Like reading a map, successfully navigating one’s way through a theory requires understanding one’s point of origin and orientation to other landmarks”. Although past research suggests that PSI characteristics may evolve over time (Boonk et al., 2018), many studies have focused narrowly on a single PSI dimension without considering the co-development of multiple, interconnected traits (Green et al., 2007; Ice & Hoover-Dempsey, 2011).

Additionally, the adaptation of instruments for use across languages and cultures is widely recognized as a complex task. Achieving equivalence—content, semantic, and conceptual—between the original and adapted versions is essential (Tsai et al., 2018). This process demands the expertise of specialists in linguistics, psychometrics, and the relevant psychological domains (Byrne, 2016). Cultural differences in how PSI is expressed behaviorally affect the way constructs are defined and measured across settings (Kristjansson et al., 2003). Our review revealed that many studies lacked a robust theoretical framework and offered limited attention to the diverse conceptual models informing family–school relations (Addi-Raccah et al., 2023).

Although the positive impact of PSI on children’s academic development is well documented (e.g., Jeynes, 2024), evidence on the relationship between PSI and academic performance remains mixed. The effect appears stronger when PSI is framed as parental expectations (Castro et al., 2015; Gubbins & Otero, 2016; Phillipson & Phillipson, 2012) or supervision and weaker—or even negative—when defined as homework assistance (Gonida & Cortina, 2014; Pezdek et al., 2002; Wilder, 2014; X. Yu et al., 2022). This variation can be attributed to differences in sample size, measurement heterogeneity, study design, and inconsistent item selection (Castro et al., 2015).

During the initial phase of this SR, qualitative PSI measures were identified. However, due to the limited validation and reliance on a few open-ended questions, only quantitative psychometric instruments were included. Psychometric tools depend on a central concept and must include sufficient items to comprehensively represent it (Kristjansson et al., 2003). Quantitative measures were chosen for their ease of administration, ability to generalize findings from large samples, and reproducibility through standardized procedures. Still, such instruments are susceptible to social desirability bias, which can affect validity (Antipkina & Ludlow, 2020; Taherdoost, 2022).

Using Bronfenbrenner’s theory as a framework, we observed that the instruments included in this SR primarily address micro-level dimensions (e.g., child or parent characteristics), without adequately incorporating macro-level influences such as educational policies or cultural systems. Creating an instrument that captures all levels of PSI is ambitious, yet necessary for a full understanding. Goldstone et al. (2023) emphasized that PSI is shaped by intersecting social, economic, and cultural forces that vary across and within nations. They advocate for more intersectional analyses that explore how race, class, gender, and cultural identity influence PSI patterns and beliefs.

Moreover, PSI should be seen as part of a larger educational and developmental framework. Parental involvement is consistently linked to children’s school belonging, academic self-concept, and emotional health (Štremfel et al., 2024), and it affects student–teacher relationships, which are crucial for motivation and adjustment (Smith et al., 2022). These impacts are most significant when PSI is viewed through an equity lens that recognizes how systemic inequalities affect family–school relationships (Rodriguez et al., 2023). Thus, PSI is not merely a predictor of academic achievement but a critical component of holistic child development.

Lerner et al. (2022) called for deeper insights into the motivations behind parental involvement, while Guo et al. (2022) recommended investigating how different forms of involvement co-evolve and how these trajectories vary across populations. Future studies should explore how PSI fluctuates over time and examine differences across age, cultural, geographic, and socioeconomic contexts.

Many studies have targeted specific populations (e.g., children with autism) or domains (e.g., math or reading). Despite our exhaustive search strategy, some relevant studies may have been missed due to overlooked search terms. Additionally, excluding gray literature may have limited the inclusion of emerging, culturally specific, or non-English instruments, potentially skewing results toward well-established tools (Paez, 2017; Schmucker et al., 2017). Future reviews may benefit from broader inclusion criteria to capture a wider range of perspectives and lesser-known instruments.

Despite its limitations, this SR offers valuable guidance for selecting PSI instruments by providing an overview of their characteristics, theoretical underpinnings, and psychometric properties.

## 5. Conclusions

The present study conducted an exhaustive analysis of instruments used to measure PSI through the perceptions of parents, children, and teachers. Additionally, we examined the theoretical models underpinning these instruments. This SR aims to support the development of consistent and replicable assessments that align with the goals of PSI-related studies while enhancing understanding of how PSI is operationalized and how reliable the resulting data are when using these tools.

Overall, this SR constitutes a relevant scientific contribution for both researchers and professionals in the field. An impactful review article not only synthesizes existing knowledge but also offers new insights and implications for policy or practice. In this regard, the findings of our study highlight critical issues in the operationalization and theoretical modeling of PSI and reveal considerable heterogeneity in how PSI is measured. This lack of consistency among PSI measures implies that interventions and policies intended to foster parental involvement in education cannot be reliably evaluated, given the absence of consensus on what the goal entails and how it should be assessed.

Therefore, this review serves as a foundation for guiding the development and empirical refinement of new evaluation instruments and contributes to broader reflections on the role of motivation in elementary school contexts. Based on our findings, we recommend that future PSI instruments prioritize strong cross-cultural validation to ensure conceptual and linguistic equivalence across diverse populations (Rodriguez et al., 2023). Moreover, adopting multi-informant approaches (i.e., gathering data from parents, children, and teachers) can offer a more comprehensive and balanced view of involvement dynamics (e.g., De Los Reyes et al., 2016).

As digital communication becomes increasingly central to family–school interactions, PSI instruments should also be updated to reflect these technological channels, such as school portals, messaging apps, and virtual parent–teacher meetings (Bordalba & Bochaca, 2019). Finally, we encourage researchers and policymakers to align PSI measurement practices with broader educational frameworks that promote inclusive, equitable, and culturally responsive engagement. To design and implement effective involvement programs, educators must understand how parents perceive their own engagement and whether their perceptions align with educators’ expectations. Therefore, measuring involvement and its barriers is both essential and urgent.

## Figures and Tables

**Table 1:** PICOST eligibility criteria.

	Parameter	Descriptor	Inclusion Criteria	Exclusion Criteria
P	Population	Parents, teachers, and children	Children aged between 6 and 15 years old and between the first and ninth grades, parents, and/or teachers	Population from kindergarten, secondary, or high school education. Children under 6 or over 15.
I	Intervention	Quantitative instruments of PSI	PSI quantitative instruments	PSI qualitative or mixed instruments; parts or subdimensions of PSI instruments or non-standard instruments
C	Comparator	NA	NA	NA
O	Outcome	Available quantitative instruments that assess PSI	Validated quantitative instruments	Non-validated quantitative instruments
S	Study design	Quantitative studies; scientific peer-reviewed articles	Scientific peer-reviewed articles in English, Portuguese, Spanish, or French	Qualitative or mixed methods. Unreviewed articles, books, theses, gray literature. Reviewed articles in other languages.
T	Time	Between 2000 and 2024	Publications between January 2000 and December 2024	Publications before January 2000 and after December 2024

Note. NA = Not applicable.

**Table 2:** Characterization of included instruments (*n* = 43).

ID	Authors (Date)	Country	Instrument	Original Authors (Date)	Subscales/Dimensions	No. Items	No. Response Questions	Adm. Length	Reading Level	Sample Size	Population
1	Pereira et al. (2003)	Portugal	Parental Involvement in School Questionnaire—teachers’ version (QEPE-VPr)	Pereira et al. (2003)	Involvement in learning activities at home/communication; school activities	24	4-point Likert scale (from strongly disagree to strongly agree)	NR	NR	121	Teachers
2	Manz et al. (2004)	USA	Family Involvement Questionnaire—Elementary (FIQ-E)	Manz et al. (2004)	Home–school communication; home-based learning; school-based involvement	46	4-point Likert scale (from rarely to always)	NR	NR	444	Parents
3	Adeyemo (2005)	Nigeria	Family Involvement Questionnaire (FIQ)	Fantuzzo et al. (2000)	Demographic questions; questions about their involvement; areas in which parents assist their children at home with general supervision of core subjects	42	4-point Likert scale	NR	NR	250	Children
4	Walker et al. (2005)	USA	Parents’ involvement scale	Walker et al. (2005)	Parents’ motivational beliefs regarding their involvement; parents’ perceptions of invitations for involvement from others; parents’ perceived life context	72	6-point Likert scale (from disagree very strongly to agree very strongly; from never to daily)	NR	NR	(a) 887 (b) 495 (c) 495	Parents
5	Walker et al. (2005)	USA	Parents’ basic involvement decisions scale	Epstein and Salinas (1993)	Home-based involvement; school-based involvement	13	Rate how likely they were to participate in a similar range of activities	NR	NR	889	Parents
6	Walker et al. (2005)	USA	Parents’ involvement forms scale	Walker et al. (2005)	Home-based involvement; school-based involvement	10	6-point Likert scale (from never to daily)	NR	NR	421	Parents
7	Solís-Cámara et al. (2007)	Mexico	Cuestionario de Participación de los Padres (CPP)/Parenting Involvement Questionnaire	Campbell et al. (1986)	Reading promotion and extracurricular activities; home based learning; personal and social child control; volunteering; pressure for achievement	30	NR	NR	NR	621	Parents
8	Pereira et al. (2008)	Portugal	Parental Involvement in School Questionnaire—parents’ version (QEPE-VPa)	Pereira et al. (2008)	Parental involvement in school activities and volunteering; family involvement in learning activities at home; school–family communication; involvement in activities at school, and participation in parents’ meetings	24	4-point Likert scale (from strongly disagree to strongly agree)	NR	NR	563	Parents
9	Olatoye and Ogunkola (2008)	Nigeria	Students’ Parental Involvement Questionnaire (SPIQ)	NR	NR	10	4-point Likert scale (from strongly agree to strongly disagree)	NR	NR	360	Children
10	Raju and Asfaw (2009)	Ethiopia	Parents’ involvement in their children’s education	NR	NR	10	3-point Likert scale and 4-point Likert scale	NR	NR	497	Children
11	Gürbüztürk and Şad (2010)	Turkey	Turkish Parental Involvement Scale (TPIS)	Gürbüztürk and Şad (2010)	Communication with teacher/school; helping with homework; personal development; volunteering; communication with child; enabling home settings; supporting personality development; supporting sociocultural development	39	5-point Likert scale (from never to always)	NR	NR	618	Parents
12	El Nokali et al. (2010)	USA	Parent–Teacher Involvement Questionnaire (parents’ version)	Miller-Johnson et al. (1995)	Parental encouragement of education; parental investment; educational attitudes	12	5-point Likert scale (from not at all to a great deal)	NR	NR	1364	Parents
13	El Nokali et al. (2010)	USA	Parent–Teacher Involvement Questionnaire (teachers’ version)	Miller-Johnson et al. (1995)	Parental encouragement of education; parental investment; educational attitudes	10	5-point Likert scale (from not at all to a great deal)	NR	NR	1364	Teachers
14	Cheung and Pomerantz (2011)	USA and China	Parents’ involvement in children’s learning	NR	NR	10	5-point Likert scale (from not at all true to very true)	NR	NR	USA: 374 China: 451	Children
15	Tekin (2011)	Turkey	Parental Role Activity Beliefs for Involvement in Children’s Education	Hoover-Dempsey and Sandler (2005)	NR	10	NR	NR	NR	374	Parents
16	Zedan (2012)	Israel	Parent Involvement Questionnaire	Toran-Kaplan (2004)	Monitoring; support and belief in the importance of studies; participation in group activities in the school and contact with the teachers; involvement when a problem arises concerning the child; participation in personal activities; indifference to the child’s achievements	35	5-point Likert scale (from very seldom to always)	NR	NR	408	Parents
17	Liu et al. (2013)	China	Perceptions of Parents Scale—child version	Grolnick et al. (1991)	Parental involvement; parental autonomy support	22	Pick statements that best describe their own parent	NR	NR	550	Children
18	Xanthacou et al. (2013)	Greece	Parental Involvement Scale	Georgiou (1999)	Homework; pressure; participation in school activities; child’s personality development; control	NR	4-point Likert scale	NR	NR	155	Parents
19	Marshall and Jackman (2015)	Barbados	Hoover-Dempsey Sandler Parental Involvement Project–Student Questionnaire	Hoover-Dempsey and Sandler (2005)	Parental involvement; student proximal academic outcomes	67	4-point Likert scale (from not true to very true)	NR	NR	155	Children
20	Fajoju et al. (2016)	Nigeria	Parental Involvement Rating Scale (PIRS)	Fantuzzo et al. (2000) and Mansfield (2009)	Four sections: A, B, C and D. Demographic questions; questions about their involvement; areas in which parents assist their children at home with general supervision of core subjects; success or failure of the pupils in the three core subjects	A = 5 B = 20 C = NR D = 3	A, B, C: Likert scale and open ended; D: success/failure	NR	NR	1895	Children
21	Kung (2016)	Taiwan	Inventory of Parental Influence (IPI)	Kung (2002)	Parental pressure; parental psychological support; parental monitoring; resources for intellectual development; parental help; parental participation in school	37	5-point Likert scale	NR	NR	363	Children
22	Ramos-Díaz et al. (2016)	Basque Country/Spain	School Engagement Measure (SEM)	Fredricks et al. (2005)	Behavioral engagement; emotional engagement; cognitive engagement	19	5-point Likert-type (from never to all the time)	NR	NR	1250	Children
23	Chen and Zhu (2017)	USA	Parent Involvement Questionnaire (PIQ)	Early Childhood Longitudinal Study—Kindergarten (ECLS-K)	Home activities and investment relevant to educational learning; family rules; involvement in school activities; communication between the school and the family; relationship between the school and the family; parents’ information network; parental attitudes toward education	25	2 items open-ended, 6 polytomous response, and 17 yes/no	NR	NR	1st grade: 15,311 3rd grade: 12,836 5th grade: 10,788	Parents
24	Pandžić et al. (2017a)	Croatia	School Engagement Measure (SEM)	Fredricks et al. (2005)	Behavioral; emotional; cognitive	19	5-point Likert scale (from never to all the time)	NR	NR	193	Children
25	Schueler et al. (2017)	USA	Family–School Engagement Scale	NR	Family–school engagement	4	5-point Likert scale (from almost never to weekly or more)	NR	NR	Study 1: 385; Study 2: 251; Study 3: 507	Parents
26	Fernández et al. (2018)	Spain	School Engagement Measure (SEM)	Fredricks et al. (2005)	Behavioral engagement; emotional engagement; cognitive engagement	19	5-point Likert scale	NR	NR	737	Children
27	Oswald et al. (2018)	USA	Parent Involvement in Children’s Learning (PICL)	McPhee et al. (2015)	Participation at school; participation in meetings; learning activities at home; learning activities in the community; parent involvement in homework	35	Yes or no	NR	NR	17,563	Parents
28	Brajša-Žganec et al. (2019)	Croatia	Parental Involvement at School scale	Sheldon and Epstein (2007)	Talk to their child’s teacher; volunteer in their child’s classroom or school; attend parental meetings; attend individual consultations with their child’s teacher; attend different school events	NR	5-point Likert scale (from never to always)	NR	NR	1024	Parents
29	Dettmers et al. (2019)	Germany	Effective Family–School Communication (EFSC)	Dettmers et al. (2019)	Regular and event independent information exchange; various forms of communication; school transitions	16	4-point Likert scale (from strongly disagree to strongly agree)	NR	NR	309	Parents
30	Veas et al. (2019)	Spain	Parent Involvement Questionnaire—Spanish version (CIF)	Veas et al. (2015)	Perception of support, organization, and interest in the educational process; expectations; school relationship; time of support with homework	20	5-point Likert scale (from never or hardly ever to always or mostly)	NR	NR	1400	Children
31	Antipkina and Ludlow (2020)	Russia	Parental Involvement SCenarios scale (PISC-9)	Antipkina and Ludlow (2020)	Home-based: learning-related activities; educational outings; school-based involvement; focus on well-being	9 scenarios	3 levels (high, medium, low)	NR	NR	1930	Parents
32	Mbaluka et al. (2021)	USA	Parent And School Survey (PASS)	Ringenberg et al. (2005)	Parenting; communicating; volunteering; learning at home; decision making; collaborating	24	5-point Likert scale, (from strongly disagree to strongly agree)	NR	NR	5144	Parents
33	Saleem and Zaffar (2021)	Pakistan	Parental Involvement Questionnaire (PIQ)	NR	Administrative; emotional; cognitive	17	Low/high	NR	NR	120	Children
34	Fisher and Refael Fanyo (2022)	Israel	Parents’ perception of parental involvement	Fisher (2011)	Improving the school’s resources; monitoring school processes; the school’s pedagogy; the school’s welfare	43	5-point Likert scale (from completely disagree to strongly agree)	NR	NR	300	Parents
35	Ji et al. (2022)	China	Parent-reported parenting involvement	Wu et al. (2015)	Interactivity, accessibility; responsibility	56	5-point Likert scale	NR	NR	1901	Parents
36	X. Yu et al. (2022)	China	Chinese version of Parental Involvement scale	Cheung and Pomerantz (2011); Grolnick and Slowiaczek (1994)	Cognitive involvement; behavioral involvement; personal involvement	23	5-point Likert scale (from never to very often)	NR	NR	234	Parents
37	Yulianti et al. (2021)	Indonesia	Parental Involvement Questionnaire (PIQ)	Yulianti et al. (2018)	Parenting; communicating; volunteering; learning at home; decision making; collaborating with community	31	4-point Likert scale	NR	NR	2151	Parents
38	Yulianti et al. (2021)	Indonesia	Teacher Invitations for Parental Involvement Questionnaire (TIPIQ)	Yulianti et al. (2018)	Parenting; communicating; volunteering; learning at home; decision making; collaborating with community	31	4-point Likert scale	NR	NR	90	Teachers
39	Goulet et al. (2023)	Canada	Student-Rated Parental School Involvement Questionnaire (SR-PSIQ)	Goulet et al. (2023)	Parental expectation; parent–child communication; homework supervision; school-based involvement	20	4-point Likert scale (from totally false to totally true)	NR	NR	923	Children
40	Cole (2024)	Jamaica	Parents’ report of parental involvement	Hoover-Dempsey and Sandler (1997)	Parents’ report of their involvement; parents’ report on invitation from teachers to be involved	12	6-point Likert scale (from never to once or more/1+ times per week)	NR	NR	210	Parents
41	Cole (2024)	Jamaica	Teachers’ report of parental involvement	Hoover-Dempsey et al. (2002)	Teachers’ beliefs about parents’ efficacy in helping their children to succeed; report of parental involvement; invitation to parents to be involved in school-related activities	29	From disagree strongly to agree very strongly; 6-point Likert scale (from never to all) 6-point Likert scale (from never to 1+ times each week)	NR	NR	24	Teachers
42	Li et al. (2024)	China	Parental involvement in child learning scale	Cheung and Pomerantz (2015)	NR	10	5-point Likert scale (from never to very often)	NR	NR	2483	Children
43	Mujtaba et al. (2024)	Tanzania	Teacher reports of parental involvement (TRPI)	Hoover-Dempsey et al. (2002)	Parents’ level of involvement	14	6-point Likert scale (from none to all)	NR	NR	169 in-service teachers	Teachers

Note. NR = Not Reported; N Response questions = Number of response questions.

**Table 3:** Psychometric characteristics of included instruments (*n* = 43).

ID	Instrument	Basis on a Theoretical Model	Inclusion of Construct Definition	Content Validity Analysis	Statistical Analysis of Items	Dimensionality Analysis (EFA or CFA)	Reliability Estimation	Evidence of External Validity		
								Criterion	Convergent	Discriminant
1	Parental Involvement in School Questionnaire—teachers’ version [QEPE-VPr]	Epstein’s model	Yes	Yes	Yes	EFA	*α* = 0.98	Yes	Yes	Yes
2	Family Involvement Questionnaire—Elementary (FIQ-E)	Epstein’s model	Yes	Yes	Yes	EFA+CFA	*α* = 0.84 to 0.91	No	No	No
3	Family Involvement Questionnaire (FIQ)	No	No	No	No	No	*α* = ≥0.70 Section A: *α* = 0.85 Section B: *α* = 0.85 Section C: *α* = 0.81	No	No	No
4	Parents’ involvement scale	Hoover-Dempsey and Sandler’s model	Yes	No	No	No	*α* = 0.62 to 0.88	No	No	No
5	Parents’ basic involvement decisions scale	Hoover-Dempsey and Sandler’s model	Yes	No	No	No	*α* = 0.89	No	No	No
6	Parents’ involvement forms scale	Hoover-Dempsey and Sandler’s model	Yes	No	No	No	*α* = 0.87 to 0.91	No	No	No
7	Parenting Involvement Questionnaire (CPP)	Epstein’s model	No	No	No	No	*α* = 0.63 to 0.88	No	No	No
8	Parental Involvement in School Questionnaire—parents’ version (QEPE-VPa)	Epstein’s model	Yes	Yes	Yes	EFA	*α* = 0.87	Yes	No	No
9	Students’ Parental Involvement Questionnaire (SPIQ)	No	No	No	No	No	*α* = 0.78	No	No	No
10	Parents’ involvement in their children’s education	No	No	No	No	No	*α* = 0.67	No	No	No
11	Turkish Parental Involvement Scale (TPIS)	No	Yes	Yes	Yes	EFA+CFA	*α* = 0.62 to 0.91	No	No	No
12	Parent–Teacher Involvement Questionnaire (parents’ version)	No	No	No	No	No	*α* = 0.85 to 0.86	No	No	No
13	Parent–Teacher Involvement Questionnaire (teachers’ version)	No	No	No	No	No	*α* = 0.89 to 0.93	No	No	No
14	Parents’ involvement in children’s learning	No	No	No	Yes	CFA	USA: *α* = 0.85 to 0.83; China: *α* = 0.83 to 0.77	No	No	No
15	Parental Role Activity Beliefs for Involvement in Children’s Education	Hoover-Dempsey and Sandler’s model	No	No	No	No	*α* = 0.79	No	No	No
16	Parent involvement questionnaire	No	No	No	Yes	CFA	*α* = 0.88	No	No	No
17	Perceptions of Parents Scale—child version	No	No	No	No	CFA	*α* = 0.57 to 0.70	No	No	No
18	Parental Involvement Scale	No	Yes	No	No	No	*α* = 0.65 to 0.84	No	No	No
19	Hoover-Dempsey Sandler Parental Involvement Project–Student Questionnaire	Hoover-Dempsey and Sandler’s model	No	No	No	No	*α* = 0.69 to 0.96	No	No	No
20	Parental Involvement Rating Scale (PIRS)	No	No	No	No	No	*α* = 0.78 to 0.82	No	No	No
21	Inventory of Parental Influence (IPI)	No	No	No	No	CFA	*α* = 0.72 to 0.85	No	No	No
22	School Engagement Measure (SEM)	No	No	Yes	Yes	CFA	*α* = 0.83 to 0.94	Yes	No	Yes
23	Parent Involvement Questionnaire (PIQ)	No	Yes	Yes	Yes	EFA+CFA	*α* = 0.53 to 0.73	No	No	No
24	School Engagement Measure (SEM)	No	No	No	No	No	*α* = 0.74 to 87	No	No	No
25	Family–School Engagement Scale	Hoover-Dempsey and Sandler’s model	Yes	Yes	Yes	CFA	Study 1: *α* = 0.89 Study 2: *α* = 0.73	No	Yes	Yes
26	School Engagement Measure (SEM)	No	No	No	No	CFA	*α* = 0.94	No	No	No
27	Parent Involvement in Children’s Learning (PICL)	No	No	No	No	No	NR	No	No	No
28	Parental Involvement at School scale	No	No	No	No	EFA	*α* = 0.66	No	No	No
29	Effective Family–School Communication (EFSC)	No	No	Yes	No	CFA	*α* = 0.91	No	No	No
30	Parent Involvement Questionnaire (CFI)	No	Yes	No	No	No	*α* = 0.65 to 0.71	No	No	No
31	Parental Involvement SCenarios scale (PISC-9)	No	Yes	Yes	Yes	Rasch rating scale model	*α* = 0.82 to 0.85	Yes	No	Yes
32	Parent And School Survey (PASS)	Epstein’s model	No	No	No	No	*α* = 0.82 to 0.85	No	No	No
33	Parental Involvement Questionnaire (PIQ)	No	No	No	No	No	*α* = 0.77	No	No	No
34	Parents’ perception of parental involvement	No	Yes	Yes	Yes	EFA+CFA	*α* = 0.90	No	No	No
35	Parent-reported parenting involvement	No	No	No	No	No	*α* = 0.94 to 0.97	No	No	No
36	Chinese version of Parental Involvement scale	No	No	No	No	CFA	ω = 0.74 to 0.82 General ω = 0.92	No	Yes * NR	No
37	Parental Involvement Questionnaire (PIQ)	Epstein’s model	No	No	No	No	*α* = 0.64 to 0.78 General *α* = 0.83	No	No	No
38	Teacher Invitations for Parental Involvement Questionnaire (TIPIQ)	Epstein’s model	No	No	No	No	*α* = 0.63 to 0.88 General *α* = 0.89	No	No	No
39	Student-Rated Parental School Involvement Questionnaire (SR-PSIQ)	No	Yes	Yes	Yes	EFA	ω = 0.66 to 0.88	Yes	Yes	Yes
40	Parents’ report of parental involvement	Hoover-Dempsey and Sandler’s model	No	No	No	No	*α* = 0.78 to 0.89	No	No	No
41	Teachers’ report of parental involvement	Hoover-Dempsey and Sandler’s model	No	No	No	No	*α* = 0.80 to 0.89	No	No	No
42	Parental involvement in child learning scale	No	No	No	No	CFA	*α* = 0.84	No	No	No
43	Teacher reports of parental involvement (TRPI)	Hoover-Dempsey and Sandler’s model	No	No	No	No	*α* = 0.70 to 0.95	No	No	No

Note. NR = Not Reported; EFA = Exploratory Factor Analysis; CFA = Confirmatory Factor Analysis; α = Cronbach’s alpha; ω = McDonald’s omega. * means convergent validity was assessed but scores were not reported.

**Table 4:** Best-rated instruments according to the identification of the theoretical model and principal psychometric characteristics.

ID	Instrument	Theoretical M	Dimensionality	Reliability	No. Items	Population	Language
1	PIS Questionnaire—teachers [QEPE-VPr]	**✓**	**✓**	**✓**	24	Teachers	Portuguese
2	Family Involvement Questionnaire—Elementary (FIQ-E)	**✓**	**✓**	**✓**	46	Parents	English
8	Parental Involvement in School Questionnaire—parents (QEPE-VPa)	**✓**	**✓**	**✓**	24	Parents	Portuguese
11	Turkish Parental Involvement Scale (TPIS)	-	**✓**	**✓**	39	Parents	Turkish and English
14	Parents’ involvement in children’s learning	-	**✓**	**✓**	10	Children	English and Chinese
16	Parent involvement questionnaire	-	**✓**	**✓**	35	Parents	English and Hebrew
17	Perceptions of Parents Scale—child version	-	**✓**	**✓**	22	Children	English and Chinese
21	Inventory of Parental Influence (IPI)	-	**✓**	**✓**	37	Parents	English and Chinese
22	School Engagement Measure (SEM)	-	**✓**	**✓**	19	Children	English and Spanish
23	Parent Involvement Questionnaire (PIQ)	-	**✓**	**✓**	25	Parents	English
25	Family–School Engagement Scale	**✓**	**✓**	**✓**	4	Parents	English
26	School Engagement Measure (SEM)	-	**✓**	**✓**	19	Children	English and Spanish
28	Parental Involvement at School scale	-	**✓**	**✓**	-	Parents	English
29	Effective Family–School Communication (EFSC)	-	**✓**	**✓**	16	Parents	English and Garman
31	Parental Involvement SCenarios scale (PISC-9)	-	**✓**	**✓**	9	Parents	English and Russian
32	Parent And School Survey (PASS)	**✓**	**✓**	**✓**	24	Parents	English
34	Parents’ perception of parental involvement	-	**✓**	**✓**	43	Parents	English and Hebrew
36	Chinese version of Parental Involvement scale	-	**✓**	**✓**	23	Parents	English and Chinese
37	Parental Involvement Questionnaire (PIQ)	**✓**	-	**✓**	31	Parents	English and Indonesian
38	Teacher Invitations for Parental Involvement Questionnaire (TIPIQ)	**✓**	-	**✓**	31	Teachers	English and Indonesian
39	Student-Rated Parental School Involvement Questionnaire (SR-PSIQ)	-	**✓**	**✓**	20	Children	English
40	Parents’ report of parental involvement	**✓**	-	**✓**	12	Parents	English
41	Teachers’ report of parental involvement	**✓**	-	**✓**	29	Teachers	English
42	Parental involvement in child learning scale	-	**✓**	**✓**	10	Children	English and Chinese
43	Teacher reports of parental involvement (TRPI)	**✓**	-	**✓**	14	Teachers	English and Swahili

*Note*. **✓** = Present.

## Data Availability

The data can be made available for consultation upon request to the corresponding author.

## Abbreviations

The following abbreviations are used in this manuscript:

PSI	Parental school involvement
SR	Systematic review
PRISMA	Preferred Reporting Items for Systematic Reviews and Meta-Analyses
MMAT	Mixed Methods Appraisal Tool

## Appendix A

**Table A1:** Information sources and search strategy.

Database	Search Strategy (Boolean Operators)	Sources	Limited Results	Expansors	Included Languages	Defined Time Range	Data Extraction
Web of Science	Parental involvement OR Parental engagement (Title) AND Measure OR Instruments OR Psychometric OR Assessment AND School AND children OR child OR teenagers OR adolescents OR parents OR mothers OR fathers OR teachers (Abstract). Also searched: Parent* involvement OR Parent* engagement (Title) AND Parent* involvement OR Parent* engagement (Abstract) OR Measure OR Instrument* OR Psychometric OR Assessment AND School AND child* OR teenag* OR adolesc* OR parent* OR mother* OR father*	Journal article	Peer reviewed	Apply equivalent subjects	English, Portuguese, Spanish, and French	From 1 January 2000 to 31 December 2024	9 January 2025
ERIC—Education Resource Information Center	Parental involvement OR Parental engagement (Title) AND Measure OR Instruments OR Psychometric OR Assessment AND School AND children OR child OR teenagers OR adolescents OR parents OR mothers OR fathers OR teachers (Abstract). Also searched: Parent* involvement OR Parent* engagement (Title) AND Parent* involvement OR Parent* engagement (Abstract) OR Measure OR Instrument* OR Psychometric OR Assessment AND School AND child* OR teenag* OR adolesc* OR parent* OR mother* OR father*	Academic journals	Peer reviewed	Apply equivalent subjects	English, Portuguese, Spanish, and French	From 1 January 2000 to 31 December 2024	9 January 2025
MEDLINE	Parental involvement OR Parental engagement (Title) AND Measure OR Instruments OR Psychometric OR Assessment AND School AND children OR child OR teenagers OR adolescents OR parents OR mothers OR fathers OR teachers (Abstract). Also searched: Parent* involvement OR Parent* engagement (Title) AND Parent* involvement OR Parent* engagement (Abstract) OR Measure OR Instrument* OR Psychometric OR Assessment AND School AND child* OR teenag* OR adolesc* OR parent* OR mother* OR father*	Academic journals			English, Portuguese, Spanish, and French	From 1 January 2000 to 31 December 2024	9 January 2025
Psychology and Behavioral Sciences Collection	Parental involvement OR Parental engagement (Title) AND Measure OR Instruments OR Psychometric OR Assessment AND School AND children OR child OR teenagers OR adolescents OR parents OR mothers OR fathers OR teachers (Abstract). Also searched: Parent* involvement OR Parent* engagement (Title) AND Parent* involvement OR Parent* engagement (Abstract) OR Measure OR Instrument* OR Psychometric OR Assessment AND School AND child* OR teenag* OR adolesc* OR parent* OR mother* OR father*	Academic journals			English, Portuguese, Spanish, and French	From 1 January 2000 to 31 December 2024	9 January 2025
PsycInfo	Parental involvement OR Parental engagement (Title) AND Measure OR Instruments OR Psychometric OR Assessment AND School AND children OR child OR teenagers OR adolescents OR parents OR mothers OR fathers OR teachers (Abstract). Also searched: Parent* involvement OR Parent* engagement (Title) AND Parent* involvement OR Parent* engagement (Abstract) OR Measure OR Instrument* OR Psychometric OR Assessment AND School AND child* OR teenag* OR adolesc* OR parent* OR mother* OR father*	Academic journals			English, Portuguese, Spanish, and French	From 1 January 2000 to 31 December 2024	9 January 2025
PsycArticles	Parental involvement OR Parental engagement (Title) AND Measure OR Instruments OR Psychometric OR Assessment AND School AND children OR child OR teenagers OR adolescents OR parents OR mothers OR fathers OR teachers (Abstract). Also searched: Parent* involvement OR Parent* engagement (Title) AND Parent* involvement OR Parent* engagement (Abstract) OR Measure OR Instrument* OR Psychometric OR Assessment AND School AND child* OR teenag* OR adolesc* OR parent* OR mother* OR father*	Academic journals			English, Portuguese, Spanish, and French	From 1 January 2000 to 31 December 2024	9 January 2025

## Appendix B

**Table A2:** Aims, main findings, strengths/limitations, and qualitative assessment of the 38 included studies.

ID	Author(s)/Publication Year	Aim(s)	Main Findings	Limitations/Strengths	Qualitative Assessment
1	Pereira et al. (2003)	This paper describes a study aimed at developing a version of a parental involvement questionnaire for teachers (p. 112).	The study of the instrument’s psychometric characteristics revealed good levels of reliability and generally supported the validity of this version (p. 128).	These instruments are necessary for a more discriminating and in-depth view of the reality of parental school involvement and can contribute to diagnosis, needs assessment, and the development of programs aimed at strengthening different forms of parental involvement (p. 129). The factor analysis of the instrument only partially supports the distinction of the three dimensions defined above (p. 128).	80%
2	Manz et al. (2004)	The purpose of this study was to replicate and extend development of the Family Involvement Questionnaire to an elementary sample of low-income urban children (p. 464).	The findings indicated that these family involvement constructs are applicable for families of preschool through fifth-grade students. Furthermore, the three dimensions comport with theoretically based multidimensional models of family involvement (p. 471).	Although the findings of this study support the preliminary development of an elementary version of the FIQ for low-income urban families, its psychometric merit is limited (p. 473). This successful preliminary study of the elementary version of the FIQ emanates from prior scholarship that broadened the conceptualization of family involvement to include multiple dimensions and established a foundation for understanding the interrelationship of family and child variables to these dimensions (p. 473).	100%
3	Adeyemo (2005)	Find out the extent to which parental involvement, interest in schooling, and school environment can impact academic self-efficacy of fresh students, and if they do, make recommendations for greater parental involvement, enhancing students’ interest and improving the school environment (p. 165).	The study further confirmed a strong link between parental involvement and academic self-efficacy (p.175).	Programs designed to improve the quality of education should place emphasis on giving students the opportunities to participate in school activities and decision making (p. 175). There is the need for policymakers to put in place an appropriate policy framework that will facilitate parents’ involvement in the education of their children (p.176).	100%
4/5/6	Walker et al. (2005)	Our primary purpose is to introduce our revised representation of psychological factors underlying parents’ involvement behaviors and describe the conceptual and methodological processes underlying their development (p. 87).	This article describes the evolution of scales designed to operationalize a theoretical model of the parental involvement process (p. 99).	Our original and revised representations of the parental involvement process offer frameworks for examining the relation between parents’ subjective involvement experiences and their actual involvement in children’s schooling. To understand how parents’ beliefs relate to their involvement behaviors, much further work is needed (p. 100).	100%
7	Solís-Cámara et al. (2007)	In this study we ask whether mothers with adequate subjective well-being, according to themselves, will show healthy parenting attitudes, in general, and also high expectations toward their children (p. 73). In this study we ask whether family income, schooling and occupation of the participants will show different distributions when comparing mothers with more adequate conditions against others (p. 73).	The findings suggest that factors other than the traditional concept of PP have differential effects on children’s academic achievement (p. 71).	This perspective does not devalue a traditional concept of PP, but on the contrary, it would allow other factors that seem more specific to the concept, such as reading promotion, to be studied to determine whether their effects are mediated by parental well-being, expectations, and parenting in general, and where other influences such as children’s own perceptions and the social influences of peers, community, and school need to be considered as well (p. 81).	100%
8	Pereira et al. (2008)	Study of the relationship between parental involvement in school, as perceived by parents and teachers, and the adjustment of primary school children (p. 91).	This study contributed to a greater understanding of the relationship between the different dimensions of parental involvement, as assessed by parents and teachers, and emotional and academic adjustment in primary school children (p. 107).	The two versions of the parental involvement in school questionnaire are not an objective measure of parental involvement (p. 106). The results relate to parental involvement in primary school and cannot be generalized to other levels of education (p. 107).	80%
9	Olatoye and Ogunkola (2008)	This study attempted to examine the probable influence of parental involvement on pupils’ achievement, especially in mathematics and science in the primary schools in Ogun State, Nigeria (p. 459).	Parental involvement is an important predictor of pupils’ achievement in science (p. 462). Parental involvement in public and private schools varies, and parents of pupils in private schools are more involved in their wards’ schooling than parents of pupils in public schools (p. 463). Male pupils enjoy more parental involvement than female pupils (p. 463).	Parents, teachers, and the school at large should overcome the age-long gender bias and come to grip with the truth that if all mediating variables are taken care of, boys and girls will both perform well alike, and so they should start to encourage both male and female pupils to be at their best so that gender is not a barrier in mathematics and science achievement (p. 463).	80%
10	Raju and Asfaw (2009)	The study aimed to find out whether test anxiety was a significant predictor of achievement of students in the presence of such variables as perceived general academic self-concept, study habits, parental involvement, and socioeconomic status among children of less-developed populations (p. 269).	Parental involvement was demonstrated in children’s study habits. It could also be concluded that parental involvement helps a child to raise his/her level of confidence and minimise the feeling of test anxiety (p. 279). Test anxiety was not found to be a predictor of achievement (…) but perceived general academic self-concept, study habits and parental involvement are better predictors of achievement (p. 280).	The significant contribution of parental involvement highlights the need for educators to recognize and incorporate into their curriculum ways to stimulate parents’ involvement at home. Schools can help enhance the ways parents supplement their child’s learning at school, such as assisting with homework or discussing what children are learning at school (p. 279). Findings underscore the importance of a two-way partnership between families and schools to best educate young children (p. 280).	80%
11	Gürbüztürk and Şad (2010)	This study mainly aimed at developing and testing the statistical validity and reliability proofs of the Turkish Parental Involvement Scale (TPIS) for the first stage of primary schools (p. 488).	Based on the findings from the study, acceptable levels of reliability and validity proofs were obtained. The Turkish Parental Involvement Scale (TPIS) is a reliable and valid scale, which can be used to define the roles, e.g., communication with teacher/school, helping with homework, etc. and levels of parents’ involvement in their children at primary school (p. 490).	It is recommended that similar studies on the validity and reliability of the scale be repeated on different populations (p. 490).	100%
12/13	El Nokali et al. (2010)	The aim of this study is to extend past research by examining within- and between-child associations among parent involvement and children’s academic and socioemotional trajectories during elementary school (p. 988).	The results of the between-child analyses suggested that higher parent involvement, as reported by mothers and teachers, promotes better social skills, fewer problem behaviors, and is unrelated to average achievement across elementary school (p. 1001).	The findings suggest that parents continue to wield considerable influence on children’s development as children progress through school. It is important for future work to explore parent behaviors that support children’s achievement (p. 1003).	100%
14	Cheung and Pomerantz (2011)	Two major goals guided the research. The first was to examine whether the nature of parents’ involvement differs in the United States and China as children progress through early adolescence. The second goal was to identify whether the effects of American and Chinese parents’ involvement on children’s adjustment during early adolescence are similar (p. 932).	The current research extends the understanding of parents’ involvement in children’s learning beyond the United States to China, where cultural ideologies about learning and parents’ role in it are different from those in the United States (p. 943).	The findings indicate that the nature of parents’ involvement differs in the two countries not only in terms of the quantity but also the quality (p. 946). Beyond issues of culture, the findings enhance the understanding of how parents’ involvement fosters children’s adjustment, suggesting that its effects on children’s engagement and achievement are unique, in that they are evident over and above the effects of parents’ control and autonomy support (p. 946).	100%
15	Tekin (2011)	Investigate parental self-efficacy for involvement in the Turkish context to contribute to a full understanding of the parent involvement process in Turkey (p. 1317).	Findings showed that Turkish parents tend to have very positive role activity beliefs (…); they believe they are able to be effective in teaching their children, and their involvement makes a positive difference (p. 1324).	This study is of great importance because it attempted to explore a subject area that has never been studied in the Turkish context. However, the study findings are based on parents’ self-reports (p. 1326). Other research, both quantitative and qualitative, is needed to detect parents’ “actual” behaviors with respect to their involvement in children’s education in order to go beyond self-reports about their beliefs and perceptions (p. 1326). Parent involvement can be achieved through effective communication between the parents and the child’s teacher (p. 1327). Finally, it is suggested that parents use school–family associations effectively to initiate more involvement activities (p. 1327).	100%
16	Zedan (2012)	The aims of this study are to measure the level of parent involvement among the Arab population in the State of Israel and to examine the relationship between the various background factors and the involvement of parents in the education of their children (p. 162).	Six relevant factors were discovered in this research: (1) monitoring (participation of the parent in the initiative of the son or daughter); (2) support and belief in the importance of learning; (3) participation in group activities in the school and contact with the teachers; (4) involvement when a problem arises with the son or daughter; (5) participation in personal activities; (6) indifference to the achievements of the son or daughter (p. 174).	Based on the belief that parental involvement has a significant impact across various populations, schools should adopt strategies to enhance parental engagement in their children’s schooling (p. 178). It is recommended that this issue be studied more intensively, both quantitatively as well as qualitatively. It might also be useful to perform confirmatory factor analysis and to apply more sophisticated statistical techniques in researching this topic, such as structural equation modeling (p. 178).	80%
17	Liu et al. (2013)	This study attempted to examine the relationship between autonomous/controlled motivation and creative thinking as well as the moderating role of parental involvement/autonomy support on this relationship (p. 446).	Results of this study showed that only specific parenting behaviors (e g., parental involvement) moderate the autonomous motivation–creative thinking link (p. 453). It is noteworthy that the moderating role of paternal involvement was different between junior and senior high school students (p. 453).	In interpreting the findings of this study, some limitations must be considered. First, doubt about the direction of causality may arise due to the cross-sectional nature of the data (p. 454). Second, rather than using fluency, flexibility, and originality scores, a composite score was employed for creative thinking tests (p. 454). Third, except for the creative thinking tests, all other measures used in this study were self-report measures (p. 454).	100%
18	Xanthacou et al. (2013)	The purpose of the study was to examine the relationship between classroom life, students’ self-esteem, anxiety, and parental involvement in primary and secondary education students. More specifically, the study aimed at examining the differences based on gender and education level in students’ classroom life as well as the level at the type of parental involvement (p. 119).	The results of the research indicate that the organization of common meetings and seminars, programs of inclusive education, where both teachers and parents are able to attend at the same time, is a major step toward a meaningful contact and exchange of views between them (p. 123).	The willingness that the parents show to become systematically involved in their children’s learning and development could lead to the groundbreaking proposal of an individual “Parents’ Curriculum”, which will direct and facilitate parents’ work at home. Thus, parental involvement and communication between parents and teachers will upgrade the cognitive, social, and emotional environment of the children in Greek schools (p. 123).	80%
19	Marshall and Jackman (2015)	This study sought to investigate the relationship between parental involvement and proximal academic outcomes as measured by active engagement. In addition, the data were examined to determine whether they provide evidence in support of the “secondary slump” phenomenon (p. 87).	The results provide compelling evidence of the nature of the relationship between parental involvement and proximal academic outcomes as measured by students’ active engagement. Firstly, the results revealed a consistent decline in students’ perceptions of parental involvement as students progress from first through third forms (p. 92).	While these findings do underscore a positive relationship between parental involvement and proximal academic outcomes, there are several variables, such as socioeconomic status, which were not included in the study but could have a moderating effect on the nature and strength of the relationship between parental involvement and proximal academic outcomes (p. 93).	100%
20	Fajoju et al. (2016)	This study investigated the relationship between parental involvement in children’s education and the academic achievement of primary six pupils in Edo State, Nigeria (p. 33). The thrust of this study was to find answers to the following questions: Will parental involvement influence pupils’ achievement in English language? Will parental involvement influence pupils’ achievement in mathematics? Will parental involvement influence pupils’ achievement in integrated science? (p. 36)	The study concludes that parental involvement significantly contributes to pupils’ achievement in English language, mathematics, and integrated science. The study further concludes that while parents focus on English as a second language, the roles of mathematics and science subjects in school should not be overlooked because mathematics and other science subjects are the bedrocks of technological development (p. 41).	An obvious limitation of this study is that the population for this study did not represent the totality of all pupils in primary schools in Edo State of Nigeria because the study was restricted to only primary six pupils. One is not likely to obtain the same result if all categories of pupils in primary school in the study area are used. Therefore, a note of caution needs to be taken when generalizing the study’s findings (p. 41).	60%
21	Kung (2016)	The purpose of the study is to examine the influence of family socioeconomic status and parental involvement on the academic achievement of middle school students in Taiwan (p.179).	The finding of the study contributed to the clarification that parents’ socioeconomic status influences children’s academic achievement both directly and indirectly via the mediating effects of parental involvement. An interesting finding concerned the only non-significant mediator, that is, that of parental pressure. It was revealed that parents’ SES did not affect the amount of pressure they put on their children (p. 185).	The study contributes to the literature in several ways. First, the study validated the six-factor model of parental involvement and helped contribute to a further understanding of the multidimensional construction of parental involvement. Second, in applying a multidimensional construction, the results showed that encouragement/psychological support was the most effective indicator for children’s academic success in Taiwan. Third, the study recognized that Taiwanese parents practiced structural/indirect involvement more than managerial/direct involvement. Fourth, social class does mediate some factors of parental involvement and both directly and indirectly influences children’s academic performance (p. 185).	100%
22	Ramos-Díaz et al. (2016)	This study’s main objective is two-fold: first, to confirm the SEM’s factor structure (Fredricks et al., 2005), and second, to analyze different types of validity and the variability between them. Regarding the second objective, there are reasons to empirically analyze variability in SEM scores as a function of level of education, because some studies have observed a drop in engagement during the transition from primary to secondary school (p. 2).	As far as concurrent validity, we confirmed that school engagement significantly influenced school performance (p. 8). By the same token, student engagement with school may have played an especially important role in the successful academic outcomes observed in this study, so it is important to develop psychoeducational interventions geared toward promoting the student’s sense of belonging and identification with school (p. 8). The educational implications derived from this study highlight the importance of promoting adjustment at school by means of an education that addresses the emotional, cognitive, and behavioral aspects of school engagement.	The educational implications derived from this study highlight the importance of promoting adjustment at school by means of an education that addresses the emotional, cognitive, and behavioral aspects of school engagement (p. 8). A limitation of this study that deserves mention is the possibility that the results presented here will vary. Validating the SEM cannot yet be considered a closed debate; as long as the questionnaire is subject to empirical testing in different, more specific sectors of the population, goodness of fit and the composition of its factors will likely vary (p. 8). Future research should test the model’s stability in different samples (for instance, at different levels of education) to establish invariance in the school engagement model (p. 8).	100%
23	Chen and Zhu (2017)	The study aimed to obtain a set of optimal items for measuring PI from kindergarten through the elementary school years and investigate whether they could be used for parents from different groups (p. 2999).	While previous studies suggested that parental involvement should include home involvement and school involvement (e.g., Bronfenbrenner 2001), the findings of this study suggest another dimension, namely, family routines. Most importantly, the results of this study suggested that the three underlying dimensions can consistently reflect PI in the middle childhood years and that the 20 items could be used to study the changes in PI from kindergarten through the elementary school years (p. 3008). The present study found that administrative time points, children’s gender, ethnicity, and SES affected the MI of a PI scale (p. 3008).	There are some limitations associated with using the ECLS-K dataset to identify the optimal items for measuring parental involvement (p. 3009). Another potential limitation might be the age of the data. Due to the limited number of items included in the present study, the findings do not demonstrate PI at a specific point in time during childhood, but are limited to kindergarten through fifth grade. Due to a lack of highly satisfactory reliability, we suggested revising binary items to polytomous items and adding items to the three domains (p. 3009).	100%
24	Pandžić et al. (2017b)	In this study, we sought to fill the gap in the literature by simultaneously examining the relationships among parental self-efficacy, parental punishment, adolescent school engagement and adolescent risky and antisocial behaviour. In other words, we examined the direct effects of parental self-efficacy on adolescent risky and antisocial behaviour as well as the serial indirect effects through parental punishment and adolescent school engagement, separately for mothers and fathers (p. 206).	The results showed that paternal, unlike maternal, self-efficacy had a direct effect on adolescents’ risky and antisocial behavior. The findings of the study point to different mechanisms by which maternal and paternal self-efficacy and adolescents’ school engagement contribute to adolescents’ risky and antisocial behavior (p. 204).	Since our proposed model is more comprehensive and includes all of the aforementioned constructs, further studies should try to replicate the results using longitudinal data and examine the potential transactional nature of this more inclusive model (p. 214). In addition, forthcoming studies should take into account the bidirectional nature of the parent-child relation. In spite of these limitations, this study represents a significant contribution to the growing literature on parenting in the context of adolescent school and socioemotional adjustment (p. 215).	80%
25	Schueler et al. (2017)	We aimed to develop tools that cover the key aspects of school-based engagement but that were short enough to ensure schools use them. Thus, this article contributes a new set of parsimonious tools that simultaneously gauge parents’ perceptions of their engagement with the school and both the school-based and out-of-school barriers that prevent greater involvement (p. 278).	Our process revealed that to effectively measure a complex construct like family engagement, researchers sometimes require a combination of scales, subscales, and composites. We illustrate that a composite variable can be useful for measuring a concept like barriers to engagement, for which the indicators measure facets of the same construct but do not function as a traditional scale (p. 297).	Our samples, on average, had somewhat higher income and education levels and were less likely to speak a language other than English at home relative to the U.S. average (p. 297). Second, these samples do not allow us to group parents by school. These findings have led to significant research and policy interest and investments in the promotion of family engagement, based on the premise that improved home–school connections should boost student achievement (p. 298). Accurate measurement of these constructs is a crucial step toward strengthening the knowledge base about the relationship between parental engagement and student achievement and about how to most effectively promote both (p. 298).	100%
26	Fernández et al. (2018)	This study has a three-fold aim: (a) to analyze the role of different parenting styles on both the school engagement and academic performance of students in compulsory secondary education and higher education (Spanish baccalaureate); (b) to determine whether it is the maternal or paternal parenting style that has a greater explanatory power for these variables; and (c) to examine which of the two dimensions that make up the parental socialization style has a greater impact on academic performance and school engagement (p. 126).	Although in this study, both maternal and paternal parenting styles seem to be more closely associated with overall affective and cognitive school engagement than with children’s school behavior, understood as both academic performance and behavioral engagement, this association is stronger in the case of paternal style (p. 133). The data obtained in this study clearly suggest that it is the level of affection and warmth and the quality of parent–child communication that is most closely associated with gaining good grades at school, while the perception of rigid control by parents gives rise to poorer grades (p. 134).	However, it may also be that the results found here are affected by the conceptualization of parenting styles, since although the structure of Maccoby and Martin’s four parenting style model (1983) was followed, dimensions such as parental criticism and rejection or inductive and indulgent discipline were not taken into account (p. 134). Future research should strive to overcome this limitation in order to determine the role played by these dimensions in relation to academic performance, school engagement, and other psychosocial adjustment variables among adolescents (p. 135).	100%
27	Oswald et al. (2018)	The goals of the study were (1) to construct a measure of parent involvement in students’ learning using data from an existing national education-related survey and (2) to investigate which child, family, and school characteristics were associated with variations in parent involvement (p. 316).	The validity of the parent involvement measure employed in the study is supported, to some extent, by the observed relationships with predictor variables (p. 321). The finding that parental involvement is greatest for students in the lower grades is unsurprising but has not been consistently reported (p. 321). The finding that parents had more involvement if their child’s health was better and less if the child had a disability is somewhat counterintuitive (p. 321). The finding that greater parent involvement with children’s learning was associated with greater satisfaction with how school staff interacts with parents is consistent with other research (p. 322).	The study reflects conceptually based decisions regarding the components of parent involvement in children’s learning and regarding how those components are weighted in a composite measure of involvement. While the method employed here shows promise in light of the findings, it is clear that other decisions might lead to different results (p. 322).	80%
28	Brajša-Žganec et al. (2019)	The aim of this study was to examine the relation of parental supervision, parental involvement at school, and children’s social competence with school achievement in primary school. A theoretical model was postulated that predicts direct and indirect effects of parental behaviors on adolescents’ school achievement (p. 1246).	The findings of this study have several practical implications for improving adolescents’ school achievement, at least among socially and academically well-functioning adolescents. Generally, although their adolescent children are showing a growing need for autonomy and independence, parents should be encouraged to remain actively involved in their children’s life in school and outside of school to positively contribute to their school achievement (p. 1255).	This study provides valuable findings regarding the relation between parental school involvement, parental supervision, and adolescents’ social competence and school achievement, but several limitations of the study should be noted (p. 1254). The study is conducted with adolescents attending primary schools in Croatia and their parents. The majority of parents were employed and had at least a high school degree, and parental school involvement and aspirations for their children’s educational attainment may vary with respect to these parental characteristics. The study used a cross-sectional design. The scales used in the assessment of parental supervision and parental school involvement showed low internal consistencies (p. 1255).	80%
29	Dettmers et al. (2019)	The aim of the present study was three-fold. Our first research question concerned the relationship between the quality of parental homework involvement and four student outcomes: achievement in mathematics and reading as well as well-being at home and school. Second, we analyzed the association between effective family-school communication (EFSC) on the one hand and parental homework involvement and the four student outcomes on the other hand. Third, we investigated the interplay between our variables, namely whether parental homework involvement mediates the association between EFSC and the four student outcomes (p. 2).	EFSC (as an indicator of FSP) may help to improve the quality of parental involvement at home, which in turn supports well-being and school achievement of students. Second, compared with the US, in Germany, much less is known about the benefits of FSP. Hence, our results of the present study hold strong importance for different groups (p. 10).	The generalization of our results is limited due to different attributes of the sample. All analyses were based on parental self-reports. Moreover, in order to improve EFSC in the school, there is a need to identify possible barriers from the school or family that may undermine teachers’ and parents’ abilities to communicate effectively with each other. The generalization of our results is limited due to the high socioeconomic status and the high proportion of mothers in our sample. The study has exclusively focused on functional ways of parenting while other parenting styles were not considered (p. 11).	100%
30	Veas et al. (2019)	The primary aim of the study is to examine and test the relations between parent involvement, metacognition, and academic achievement in early adolescence to gain a deeper understanding of these constructs (p. 398).	The mediating effect of metacognitive strategies in the relation between parent involvement and academic achievement both at the student level (L1) and class level (L2) allows us to confirm two important findings (p. 404) First, both at the group and individual level, social interactions between parents and children are necessary for academic success. Moreover, as the difficulty of learning increases in secondary education, together with psychological changes during adolescence, students need to fulfil their personal goals (p. 405).	The present study highlights the importance of metacognition during early adolescence, apart from other self-regulatory or cognitive processes (p. 405). The classification of students with high and low achievement should be performed in future studies to observe different influence patterns of the target variables as a function of achievement (p. 405). There is not a generalized validated scale in Spain that measures parent involvement in adolescence. This study did not obtain the reporters’ views of parents (p. 405).	100%
31	Antipkina and Ludlow (2020)	This study describes a Rasch–Guttman scenario-based scale designed to provide a holistic approach to measuring the PI construct (p. 847).	This article describes the development of the PISC-9. The development of this scale was prompted by concerns that the construct of parental involvement allows for multiple definitions and operationalizations and is often too narrowly focused on specific aspects of parental involvement (p. 860). In contrast to such an approach, we have shown that a scale based on Rasch measurement principles and Guttman facet theory design can measure a range of authentic PI behaviors (p. 860).	The scenario format may be more demanding of the reading and language skills of respondents than traditional short-stemmed Likert-type items (p. 862). Despite the fact that the scenario format is more demanding to employ than traditional item and scale development procedures, it allows for a flexible definition of the construct (p. 862).	100%
32	Mbaluka et al. (2021)	The purpose of this study was to investigate whether students’ self-discipline and parental involvement in students’ academic activities have any impact on students’ Iowa Test of Basic Skills (ITBS) scores or on their GPA (p. 270).	The findings of this study reveal that student self-discipline and parental involvement are crucial factors in student academic performance (p. 289). Parental involvement was highly correlated with GPA and ITBS performance. Combined, student self-discipline and parental involvement displayed significant impact on academic performance. Findings indicated that in order to improve GPA and ITBS performance, parents need to participate actively in the academic activities of their children, including communicating with the school, parenting, volunteering, decision making, facilitating learning at home, and collaborating with the community to provide resources to support schools (p. 290).	Pilot study: 1. The initial pilot study had only 16 participants. 2. Socioeconomic status (SES) was not included as a demographic variable because most Adventist schools do not collect SES data from parents (p. 290). Primary study: 1. Out of the six factors in Epstein’s parental involvement model, only four could be tested in this study: communicating, parenting, volunteering, and learning at home. The other two constructs, decision making and collaborating with the community, could not be evaluated in the primary study because the CG data used for the primary study had insufficient data on decision making and collaborating. 2. Again, socioeconomic status (SES) was not investigated with other demographic variables because most Adventist schools do not collect SES data from parents (p. 290).	100%
33	Saleem and Zaffar (2021)	To examine the multiple facets of parental involvement among the parents of primary-level students and to compare the effect of multiple facets of parental involvement on the homework performance of primary-level students (p. 61).	The results of this study provide strong evidence that parental involvement in homework assistance in various forms, such as overall environment of the home, monitoring of homework by parents, reading out lessons to children, and helping to solve difficult questions have excellent impact on the homework performance of their children. Parental involvement therefore confirms the significance of involving parents in homework activities, especially regarding the facets of cognitive and administrative involvement (p. 73).	Parents may fix specific times and provide a specific place to their children during homework activities to obtain better administrative control over the activities. Parents may check the homework dairy of their children daily (p. 73). There must be a mutual collaboration between institutes and parents, so that problems regarding children’s homework activities can be easily dealt with. Seminars and workshops may be organized for the parents to emphasize their role of managing activities that can elevate children’s academic achievements (p. 73).	60%
34	Fisher and Refael Fanyo (2022)	The study aimed to gauge the effect of the level of communality on the way parents of children enrolled in government-funded elementary schools perceive the concepts of teacher authority and parental involvement (p. 5).	The analysis revealed that parents perceived parental involvement as containing the following four components: monitoring of processes at school, supporting school resources, awareness of pedagogical methods within the school, and active participation in these processes (p.9). A significant finding that emerged from the structural equation analysis was a strong relationship between parents’ perception of teachers’ authority and their perception of parental involvement (p. 10).	In terms of its practical applications, the model can help education systems in general, and schools in particular, to formulate policies and take steps to improve the ever-important relationship between the school and the parents (p. 1). Furthermore, the model clarifies our understanding of and ways to strengthen the teacher’s authority (p. 1).	100%
35	Ji et al. (2022)	This study investigated the relationship between maternal positive coparenting and adolescent peer attachment and the intermediary role of parental involvement and parent–child attachment (p. 01).	Parental involvement and parent–child attachment played a significant mediating role between maternal positive coparenting behavior, including unity and consistent behavior, and adolescent peer attachment, respectively, which consisted of a sole intermediary role of parental involvement; a single intermediary role of parent–children attachment; and a chain intermediary role of parental involvement and parent–children attachment (p. 01).	This study contributes to our understanding of the chain mediating processes in the association between maternal positive coparenting behavior and adolescent peer attachment. Based on this exploratory approach, this study examined a mediation model emphasizing the role of mothers’ involvement and parent–child attachment and found that parental involvement and parent–child attachment play a significant mediating role on the associations between mothers’ positive coparenting behavior and peer attachment through three specific paths: an independent mediating role of parental involvement, an independent mediating role of parent–child attachment, and a chain mediating role of parental investment and parent–child attachment (p. 08).	80%
36	X. Yu et al. (2022)	The present study aimed to examined the associations among parental involvement, children’s learning engagement, parental psychological control, and children’s academic achievement during the COVID-19 pandemic (p. 1625).	The current study provided some of the earliest evidence on the relationship between parental involvement and children’s academic performance during school closures caused by COVID-19 (p. 1633). Specifically, the quality of parental involvement and children’s learning engagement were linked to children’s academic achievement during school closure. Parental psychological control moderates the association between parental involvement and children’s learning engagement. The present findings provided important implications for parents and educators. Specifically, due to the lack of support from teachers and peers, the supportive role of parents becomes prominent for promoting children’s academic outcomes during school lockdown (p. 1633).	The current study has many strengths, such as its focus on the associations between parental factors and children’s academic achievement and the recruitment of three-wave data. Notably, the current study also had several limitations. First, the self-report questionnaires were used for all measures except children’s academic achievement measures, which might result in information bias. Second, China controlled the COVID-19 outbreak quickly, and the situation returned to normal soon after the occurrence of COVID-19, which is different from many other countries. Thus, the generalizability of the current findings should be further tested in other countries. Third, it needs to be acknowledged that the relationship between parental involvement and children’s academic outcome was correlational, which could not obtain casual inferences from the present model (p. 1633).	100%
37/38	Yulianti et al. (2022)	In this study, we aimed to study the relationships of transformational leadership for parental involvement and teacher invitations with parental involvement practices (p. 101).	This study provides insights into how transformational leadership for parental involvement and teacher invitations for parental involvement may promote parental involvement practices, particularly in Java, Indonesia. Transformational leadership did not have significant effects on parental involvement. However, there were some significant effects of teacher invitations on parental involvement which may be targeted by transformational leadership practices in their school (p. 110).	This study also has limitations. With respect to the surveys on transformational leadership for parental involvement and teacher invitations for parental involvement, we relied on teachers’ perceptions and teachers’ self-reports. Another limitation is the cross-sectional nature of this study (p. 110). Professional development for teachers should be aimed at fostering their professional competence in establishing relationship with parents and inviting parents to be involved in their children’s education. At government level, there should be sufficient budget to provide such education programs for school leaders, teachers and parents (p. 110).	100%
39	Goulet et al. (2023)	The aim of the present study was to undertake the preliminary validation of the Student-Rated Parental School Involvement Questionnaire (SR-PSIQ) (p. 419).	This study is one of the rare studies that tested factorial validity, measurement invariance, and predictive validity of students’ perception of parental involvement among a sample of young elementary school children. Our results support the multidimensionality of parental involvement (p. 427).	While the SR-PSIQ seems to be a valid instrument that can be used among different populations, the present sample came from a highly disadvantaged urban area. Even if factorial structure withholds, other differences could occur among populations with higher SES. Namely, the negative link between school-based involvement and engagement may be an artifact of parents’ low SES (p. 428). Given that the SR-PSIQ reflects students’ perception of parental involvement, certain aspects are not taken into consideration. Nevertheless, these dimensions remain important to fully understand parental involvement, and well-developed and validated tools assessing parents’ or teachers’ points of view are available if these aspects are of interest (p. 429).	100%
40/41	Cole (2024)	This cross-sectional research investigates the extent to which school locations influence parental involvement and the cognitive skills of students in the first grade (p. 1).	The early and continued involvement of parents in their children’s education in rural, city, and inner-city schools influences students’ outcomes. Invitations to be involved and teachers’ reports of parental involvement are minimally moderated by the location of schools. Parents who are involved are least likely to be invited to be involved, and the students are more likely to have higher cognitive skills. The findings suggest that schools located in rural communities may be under-resourced, and parents may require more resources and skills to help their children (p. 13).	Relying on the parental involvement model may not capture all the components that could impact parental involvement. In addition, because the data were collected from parents’ and teachers’ self-reports of parental involvement, it could be skewed as a result of their interpretations. The findings of the research cannot be generalized based on the sampling method. The sample size was not equal across groups, which may affect the comparison across groups. The sex of students and parents and their socioeconomic status were not investigated. This could influence parental involvement and students’ cognitive skills (p. 13).	100%
42	Li et al. (2024)	This study aimed to identify the developmental trajectories of the subjective well-being of adolescents in early adolescence. The aims were to determine whether gender and parental involvement can predict different developmental trajectories. Subgroups that differed in their typical developmental patterns and the effects of predictors were examined to provide effective prevention and intervention strategies for the subjective well-being of adolescents (p. 736).	This study identified different heterogeneous patterns of Chinese adolescents, implying various development types of subjective well-being in adolescence from a hedonistic perspective. Individual-centered GMM analysis was performed to offer information on the dynamic process of the samples. Furthermore, gender and parental involvement were considered to examine their effects on adolescents’ developmental trajectories of subjective well-being (p. 742).	Despite its important theoretical and practical implications, the limitations should be considered. First, the present study was a longitudinal design, but the follow-up time was limited, and only seventh-grade students in middle China participated. Due to age and time limitations, it is impossible to comprehensively describe the subjective well-being of individuals throughout the whole adolescence period. Further studies are needed to extend the sample and tracking time. Second, “the low-decline group”, as an at-risk group of the subjective well-being development trajectory, needs more in-depth research to explore its profiles and characteristics to heighten their subjective well-being level. In addition, this study examined only the influence of gender and parental involvement factors on the developmental trajectories of the subjective well-being of individuals (p. 744).	80%
43	Mujtaba et al. (2024)	The aim of this study is to evaluate the effect of interventions on the beliefs of pre-service and in-service teachers regarding parental involvement within the specific context of rural Tanzania. The study is guided by the question: Can a capacity-building teacher training program to enhance parental involvement lead to increased teacher beliefs and reports on parental involvement? (p. 3)	For pre-service teachers, only beliefs on the importance of specific involvement practices were measured, which showed an increase over time (p. 6). For in-service teachers, overall results show that self-reported beliefs regarding the importance of specific involvement practices and parent efficacy for helping children at school were significantly enhanced due to the teacher training program (p. 6).	A strength of this study was that the in-service teacher intervention took place in a real environment within regular day-to-day activities, increasing the external validity of the findings. In general, the longitudinal design with three measurement occasions (baseline, second measurement, and follow-up) was also a strength since not only the immediate, but also the longer-term effects of the intervention could be measured (p. 7). It was limited by the fact that the intervention was assigned to schools rather than individuals within schools. The study is also limited since a control group was not included for the pre-service teacher intervention. Reports of invitations to parental involvement as well as reports of parental involvement were provided by teachers themselves, leaving the possibility of biased responses. It is also limited because the questions were not all directly linked to the content of the training (p. 7).	100%
